# Amine Functionalized Gadolinium Oxide Nanoparticles-Based Electrochemical Immunosensor for Cholera

**DOI:** 10.3390/bios13020177

**Published:** 2023-01-23

**Authors:** Ashutosh Kumar, Tamal Sarkar, Pratima R. Solanki

**Affiliations:** 1 Department of Electrical Engineering, University of Notre Dame, Notre Dame, IN 46637, USA; 2 Department of Physics, Indian Institute of Technology Kharagpur, Kharagpur 721302, India; 3 Special Centre for Nanoscience, Jawaharlal Nehru University, New Delhi 110067, India

**Keywords:** gadolinium oxide nanoparticles, APTES, immunosensor, *Vibrio cholerae*

## Abstract

Herein, we report the synthesis and functionalization of gadolinium oxide nanoparticles (Gd_2_O_3_ NPs) to fabricate a highly efficient immunosensor for the detection of *Vibrio cholera* toxin (CT). Gd_2_O_3_ NPs were produced in a straightforward manner utilizing the microwave irradiation technique using a domestic microwave oven. X-ray diffraction, transmission electron microscopy, and spectroscopic techniques were used to characterize the structural and physical aspects of Gd_2_O_3_ NPs. The Gd_2_O_3_ NPs were then functionalized with 3-(Aminopropyl) triethoxysilane (APTES) and electrophoretically deposited onto an ITO-coated glass substrate. The anti-CT monoclonal antibodies were covalently attached to the APTES-Gd_2_O_3_/ITO electrode via EDC-NHS chemistry, followed by bovine serum albumin (BSA). For CT detection, electrochemical response experiments using BSA/anti-CT/APTES-Gd_2_O_3_/ITO immunoelectrodes were carried out (5–700 ng mL^−1^). The immunoelectrode demonstrated an outstanding electrochemical reaction against CT, with a sensitivity of 8.37 mA ng^−1^ mL cm^−2^ and a detection limit of 1.48 ng mL^−1^.

## 1. Introduction

The development of cutting-edge diagnostic instruments and novel treatment agents is now possible due to interdisciplinary research on nanomaterials and their composites [1]. Recently, the amalgamation of nanomaterials in interdisciplinary investigations has had a substantial effect on probable biomedical uses, involving bioimaging, biosensing, and targeted drug delivery [2,3]. Nowadays, metal oxide nanoparticles (NPs) have been designed to create significant nanomaterials, which deliver an operative surface for the immobilization of biomolecules [4,5]. Gadolinium oxide (Gd_2_O_3_) NPs have recently been proven to be non-toxic, compatible with biological analogs, andexhibit catalytic qualities, and they can also be manufactured into intriguing morphological shapes at the nano level [6]. Gd_2_O_3_ NPs exist in the following three crystallographic forms: hexagonal, monoclinic and cubic due to their thermal, chemical stabilities, rigid emission and wide band gap of 5.3 eV [7,8]. Gd_2_O_3_ NPs can be synthesized using various methods, including thermal decomposition, hydrothermal, chemical precipitation, sol-gel, reflux and microwave irradiation [9,10]. Among these methods, microwave synthesis of NPs is favorable because of its rapid chemical reactions, lowertime consumption, clean chemistry, better yield and reproducibility as compared to hydrothermal methods [11]. In the last decade, researchers have extensively explored Gd_2_O_3_ NPs for diverse biomedical applications including magnetic resonance imaging, targeted delivery, and nuclear medicine [12]. Gadolinium has maximum unpaired electrons in its ionic state; therefore, Gd_2_O_3_ NPs may possess outstanding electrochemical properties [13]. Although there are a few reports of the application of Gd_2_O_3_ NPs in sensor fabrication, it is still an unexplored material for sensor development.

Diarrheal cholera is a life-threatening disease worldwide and has a major impact on developing countries. Gram-negative bacteria from the *Vibrionaceae* family are the leading cause of diarrheal cholera [14]. *V*. *cholerae* O1 and O139 serotypes produce cholera toxin (CT), which is the cause of infection. CT-contaminated water and food are the most common mode of cholera spread. CT is an oligomeric protein composed of homopentameric and heterodimeric subunits. After entering the host, CT leads to massive fluid secretion which leads to diarrhea [15]. For the diagnosis and prevention of cholera, major developments have been made during the last decade, including co-agglutination, enzyme-linked immunosorbent assay, bioassays using animal tissue culture and conventional culture-based assays [16]. These techniques require sophisticated instruments, manpower, higher costs, and long assay times. Therefore, biosensors are the best solution for fast response times, cost-effectiveness and highly specific detection. The detection of CT has been reported by various research groups using electrochemical immunosensors. Patel et al. used a monolayer of thiolated ssDNA on the gold electrode to hybridize genomic DNA (ds DNA/Au) for the detection of *Vibrio cholerae* (detection range 100–500 ng mL^−1^) with a sensitivity of 0.027 μA ng mL^−1^ cm^−2^ and a limit of detection (LOD) of 100 ng mL^−1^ [17]. Solanki et al. fabricated a *Vibrio cholerae* immunosensor based on reduced graphene oxide (RGO) on anatase TiO_2_ nanohybrid with a sensitivity of 21.8 × 10^−3^ μF ng mL^−1^ cm^−2^, LOD of 0.15 ng mL^−1^ in the detection range of 10–450 ng mL^−1^ [18]. Sharma et al. used biocompatible citric acid-capped magnetite NPs for CT (12.5–500 ng mL^−1^) detection with a sensitivity of 0.03 Ω ng mL^−1^ cm^−2^ and LOD of 0.32 ng mL^−1^ [19]. Bagbi et al. reported the results of the CT immunosensor based on a nanocomposite of zirconium oxide NPs and gelatin with a sensitivity of 0.03 Ω ng mL^−1^ cm^−2^ and LOD of 0.74 ng mL^−1^ in a detection range of 50–400 ng mL^−1^ [20].

In this work, a simple, efficient, and label-free biosensing platform based on nanostructured Gd_2_O_3_ NPs was fabricated for CT detection. The microwave irradiation technique was used to synthesize monodispersed Gd_2_O_3_ NPs. Amine (–NH_2_) functionalization of Gd_2_O_3_ NPs was achieved using the3-(Aminopropyl) triethoxysaline (APTES) linker molecule. APTES-Gd_2_O_3_ NPs were deposited onto an indium-tin-oxide (ITO)-coated glass substrate via the electrophoretic deposition method, followed by the covalent immobilization of antibodies specific to CT (anti-CT) and BSA. The cellular interaction of Gd_2_O_3_ NPs was studied in RAW 264.7 cells. This is the first report where Gd_2_O_3_ NPs were applied for electrochemical sensing for CT recognition.

## 2. Materials and Methods

### 2.1. Materials

Acetonitrile anhydrous 99.8% (CH_3_CN), potassium ferricyanide (K_3_[Fe(CN)_6_]), potassium ferrocyanide (K_4_[Fe(CN)_6_], 3H_2_O) and sodium hydroxide (NaOH) were bought from Fisher Scientific, India. APTES, 1-(3-(dimethylamino)-propyl)-3-ethyl carbodiimide hydrochloride (EDC) and N-hydroxysuccinimide (NHS) were purchased from Sigma-Aldrich, Germany. Gadolinium (III) nitrate hexahydrate [Gd (NO_3_)_3_, 6H_2_O] and sodium chloride (NaCl) were procured from CDH, India. Monoclonal antibodies specific to cholera toxin (anti-CT), bovine serum albumin (BSA) and *Vibrio cholerae* toxins (CT) antigens were purchased from M/s Genetix Asia Pvt. Ltd. All of the chemicals were of an analytical grade and were put to use without any additional purification.

### 2.2. Synthesis of Gd_2_O_3_ NPs

Gd_2_O_3_ NPs were synthesized using a one-step microwave irradiation technique. A 1.89 g gadolinium nitrate solution was dispersed in 50 mL of DI water and stirred for 30 min at 60 °C; then, 0.5 M NaOH was added into the prepared solution while stirring until the pH of the solution reached10 for 3 h at 60 °C. After this, the solution was microwaved at 900 W for 100 s using a household microwave oven (LG 1350-Watt 2450 MHz). Once the reaction was complete, the vessel was left to cool to room temperature. The solution was washed with DI water five times, followed by ethanol washing using the centrifugation process 5–6 times or until the pH of the solution became neutral. The slurry was dried at 80 °C overnight. The dried product was annealed at 600 °C for 3 h. Finally, the product was crushed into a fine powder using a mortar and pestle for advanced characterizations.

### 2.3. Functionalization of Gd_2_O_3_ NPs with APTES

**Scheme 1:**
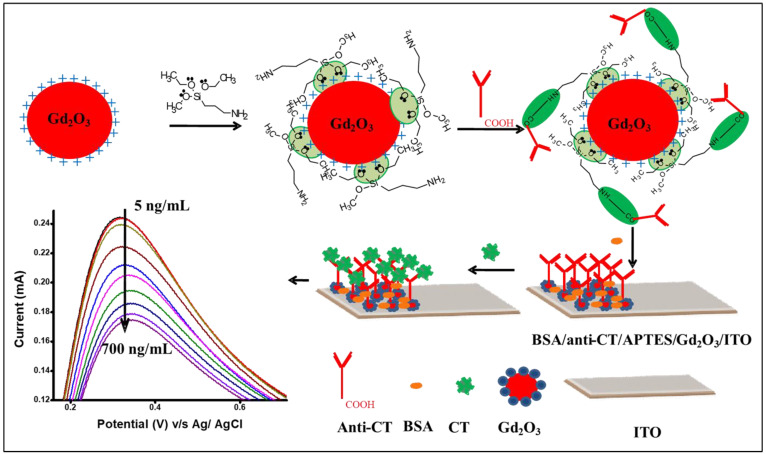
APTES-functionalized Gd_2_O_3_ NPs and preparation of BSA/anti-CT/APTES/Gd_2_O_3_/ITO with response study of immunoelectrode as a function of CT.

We dissolved 100 mg of synthesized Gd_2_O_3_ NPs in 150 mL of propan-2-ol and performed sonication (15 min), followed by stirring at 300 rpm at 55 °C to obtain a highly dispersed suspension. We then mixed 1 mL of APTES (0.946 gm mL^−1^) into the prepared solution with dropwise addition, generating a large number of amines (–NH_2_) groups on the surface of Gd_2_O_3_ NPs. Positively charged [21] Gd_2_O_3_ NPs easily bonded with the oxygen of APTES via covalent coordinate bond formation [22] (Scheme 1). Finally, 75 mL of DI water was added to the above solution. The prepared solution was stirred at 300 rpm for 24 h at 55 °C. After 24 h, the mixture was filtered and washed several times with DI water to remove unbound APTES molecules. Subsequently, the obtained slurry (APTES-Gd_2_O_3_NPs) was dried at 50 °C for 48 h, yielding whitish material [23].

### 2.4. Electrophoretic Deposition of APTES-Gd_2_O_3_ NPs onto ITO-Coated Glass Substrate

APTES-Gd_2_O_3_ NPs were deposited on a pre-hydrolyzed ITO-coated glass substrate using the electrophoretic deposition (EPD) technique. Before deposition, a colloidal suspension of 5 mg of APTES-Gd_2_O_3_ NPs was made in 0.5 mL of acetonitrile and 0.5 mL of ethanol followed by ultrasonication (30 min) at room temperature. For the electrophoretic deposition process, a DC voltage source was used, and two electrode systems were employed. The surface charges on APTES-Gd_2_O_3_ NPs were enhanced by mixing magnesium nitrate into the above colloidal suspension that acted as an electrolyte. Platinum foil and pre-hydrolyzed ITO glass were used as a counter and the working electrode, respectively. The electrodes were placed at a separation of 1 cm and were immersed into the 3 mL solution. A DC voltage of 60 V was applied for 90s to deposit a uniform film of APTES-Gd_2_O_3_ NPs on the ITO glass substrate (APTES-Gd_2_O_3_/ITO). The surface area of the APTES-Gd_2_O_3_/ITO electrode was maintained at 0.25 cm. After being deposited, the electrodes were extracted from the solution and cleaned with DI water before being dried overnight at room temperature (25 °C).

### 2.5. Immobilization of Anti-CT onto APTES-Gd_2_O_3_/ITO Electrode

Antibody specific to cholera toxin (anti-CT) solution (100 µg mL^−1^) was freshly made in PBS (pH 7.0). A total of 20 µL of the anti-CT solution, EDC (0.2 M) and NHS (0.05 M) were mixed in a volumetric ratio of 2:1:1, kept for 30 min and then drop cast onto the APTES-Gd_2_O_3_/ITO electrode. After letting the electrode rest in a humidified atmosphere at ambient temperature for 6 h, we rinsed it with phosphate-buffered saline (pH 7.0) to remove any stray antibody molecules. The carboxylic group (-COOH) of anti-CT (Fc region) covalently tied with the –NH_2_ terminal of APTES-Gd_2_O_3_ NPs via a solid amide bond (CO-NH) that was later validated by IR studies. Then, BSA (0.1 wt%) was employed to inhibit the electrode’s nonspecific reactive groups. Before being stored at 4°C, the BSA/anti-CT/APTES-Gd_2_O_3_/ITO immunoelectrode was washed with PBS. Scheme 1 shows the various steps for the fabrication of a working immunoelectrode.

### 2.6. Characterizations

Using a powder X-ray diffractometer (Rigaku MiniFlex 300), we were able to ascertain the degree of crystallinity of the Gd_2_O_3_ NPs. The XRD analysis was carried out at 2θ Bragg’s angle, with the range fixed at 30–80 degrees and the scan rate set at 20 degrees per minute. Moreover, FE-TEM was used to conduct shape, size, dispersity and electron diffraction investigations of Gd_2_O_3_ NPs (JEOL JEM-2100F TEM). After overnight drying at 37 °C, a sample was made by drop-casting a solution of well-dispersed Gd_2_O_3_ NPs in ethanol onto a carbon-coated copper grid (300 mesh). In order to determine the optical properties of the Gd_2_O_3_ NPs, ultraviolet-visible absorption spectroscopy was carried out on a T90+ UV/VISspectrometer while suspending the Gd_2_O_3_ NPs in DI water. Furthermore, to check the emission spectrophotometric properties, the fluorescence spectrumwas obtained with a Cary Eclipse fluorescence spectrophotometer. Moreover, to study the chemical interactions occurring during various steps of immunoelectrode fabrication, Fourier transform infrared (FTIR) spectroscopy was carried out on a Varian 7000 FTIR spectrometer. To check the changes in the physical morphologies of electrodes during anti-CT immobilization, scanning electron microscopy (SEM) was performed by using a Zeiss EVO 40 microscope. The electrochemical characterizations and responses were carried out using AMETEK PARSTAT Potentiostat/Galvanostat, which has a three-electrode system including a working electrode, an Ag/AgCl electrode as a reference electrode and a counter electrode of platinum foil. A phosphate buffer saline (PBS pH 7.0 and 0.9% NaCl) solution containing 5 mM [Fe (CN)_6_]^3−/−4−^ was used as an electrolyte.

## 3. Results and Discussion

### 3.1. Structural and Morphological Study

**Figure 1:**
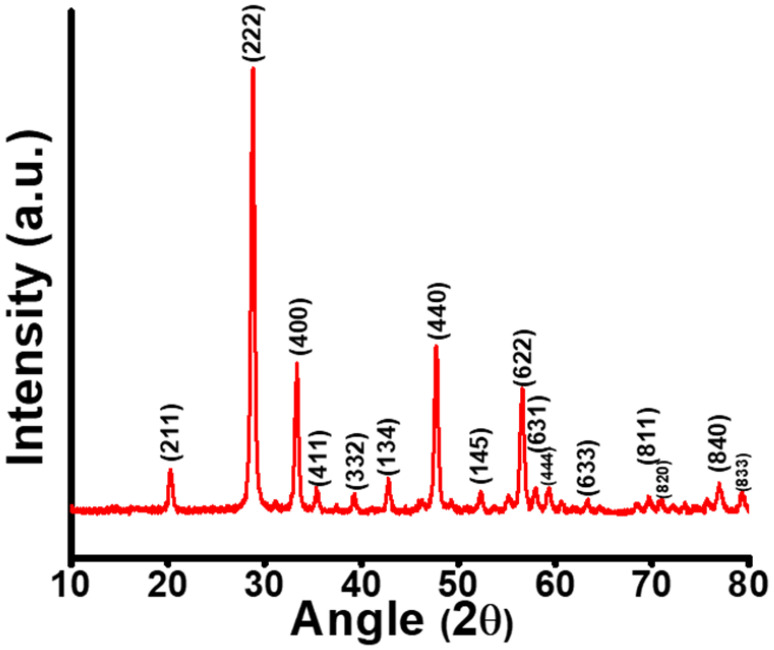
X-ray diffraction pattern of Gd_2_O_3_ NPs.

The X-ray diffraction pattern of Gd_2_O_3_ NPs (Figure 1)depicts the diffracted peaks corresponding to the (211), (222), (400), (411), (322), (134), (440), (145), (622), (631), (444), (633), (811), (820), (840) and (833) planes, which were well indexed with JCPDS No: 43-. This X-ray diffraction pattern revealed the formation of a pure cubic phase of Gd_2_O_3_ NPs [10]. The average crystallite size was estimated to be 19 (±2) nm considering all peaks by using the Debye-Scherrer equation as follows:

(1) $$D=\frac{0.9\lambda }{\beta cos\theta }$$

where *λ* = 1.5460 Å is the wavelength of target Cu-Kα, *θ* is the Bragg’s angle of diffraction and *β* is the full-width half maximum of diffraction peak.

**Figure 2:**
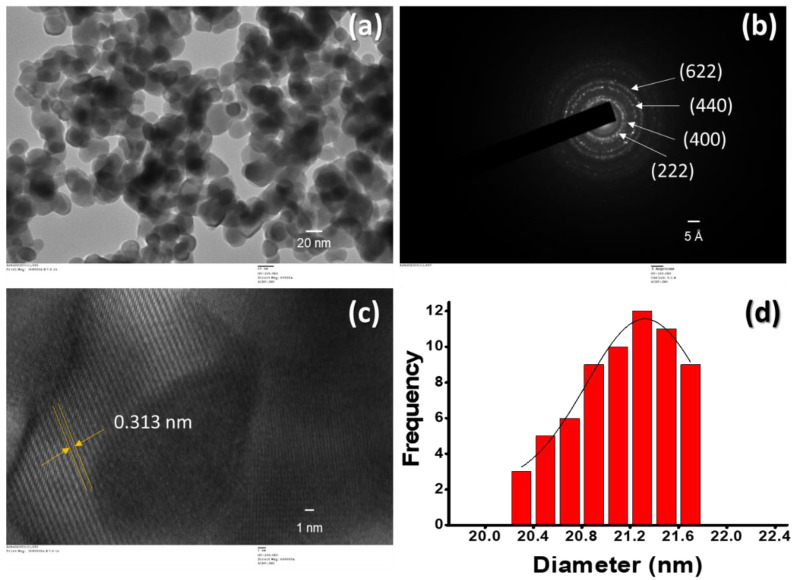
HR-TEM images of (**a**) Gd_2_O_3_ NPs; (**b**) SAED pattern of Gd_2_O_3_ NPs; (**c**) HR TEM images of Gd_2_O_3_ NPs; and (**d**) NPs size distribution.

TEM images show the structure and morphology of Gd_2_O_3_ NPs. Figure 2a shows the formation of quasi-spherically shaped and mono-dispersed Gd_2_O_3_ NPs. The average obtained size of NPs was between 20 and 22 nm, which was in agreement with the crystalline size estimated by XRD. The selected area electron diffraction (SAED) pattern shows bright lattice fringes, which were assigned to (222), (400), (440) and (622) planes (Figure 2b). It was anticipated that there was a formation of polycrystalline Gd_2_O_3_ NPs. Figure 2c shows that the HRTEM led to a d spacing of about 0.313 nm, which directly corresponds to the XRD plane (222). The nanoparticle size distribution varied in the range of 20.3 to 21.7 nm per image (Figure 2d). All HRTEM results were in good agreement with the XRD data, indicating the formation of monodispersed and polycrystalline Gd_2_O_3_ NPs.

**Figure 3:**
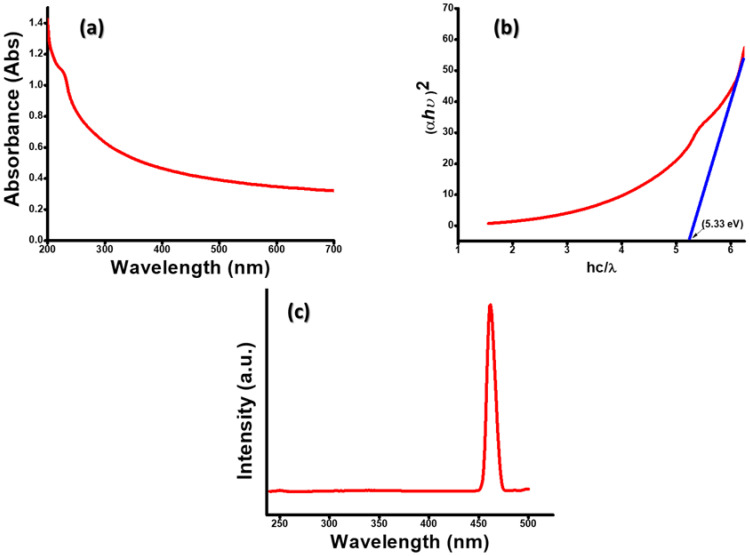
(**a**) UV/Vis spectrum; (**b**) Tauc’s plot; (**c**) fluorescence spectrum of Gd_2_O_3_ NPs.

Figure 3 shows the UV-Vis spectroscopic study of Gd_2_O_3_NPs, which was carried out using DI water as a solvent. The absorption peak was found at 230 nm (Figure 3a). Using Tauc’s equation, the direct band gap of Gd_2_O_3_ NPs was determined. A plot between the (*αhυ*)^2^ and band energy, where *α* is absorbance, *h* is the plank constant, *υ* is frequency and *k* is a constant (Figure 3b). The direct band gap was calculated to be 5.33 eV, which was higher than the bulk materials.

(2) $${\left(\alpha h\nu \right)}^{2}=k\left(h\nu -E_{g}\right)$$

Moreover, Gd_2_O_3_ is well known for its optical properties, as it is an ideal host for photoluminescence. Gd_2_O_3_ NPs showed very high fluorescence at 462 nm when excited by a230 nm wavelength (Figure 3c). The peak maxima near 462 nm could be attributed to surface defects on NPs of Schottky and Frenkel type [24].

**Figure 4:**
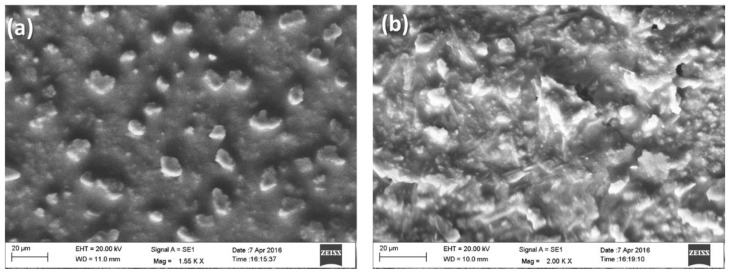
SEM images of (**a**) APTES/Gd_2_O_3_/ITO; (**b**) anti-CT/APTES/Gd_2_O_3_/ITO electrodes.

The surface morphology of the (a) APTES-Gd_2_O_3_/ITO electrode and (b) anti-CT/APTES-Gd_2_O_3_/ITO electrode is shown in Figure 4. Figure 4a shows the uniform deposition of sub-micrometer size NPs on the electrode surface. The uniform deposition could be due to the accumulation of NPs during electrophoretic deposition. While making the uniform film with electrophoretic deposition over the ITO electrode, NPs attached to each other and could be seen as sub-micron particles. Moreover, after the immobilization of anti-CT and BSA onto APTES-Gd_2_O_3_/ITO electrode surface (Figure 4b), the morphology of the electrode completely changed into fiber-like structures around the NPs, which indicated the immobilization of anti-CT on to the APTES-Gd_2_O_3_/ITO electrode. The immobilization of anti-CT was further confirmed using FTIR.

**Figure 5:**
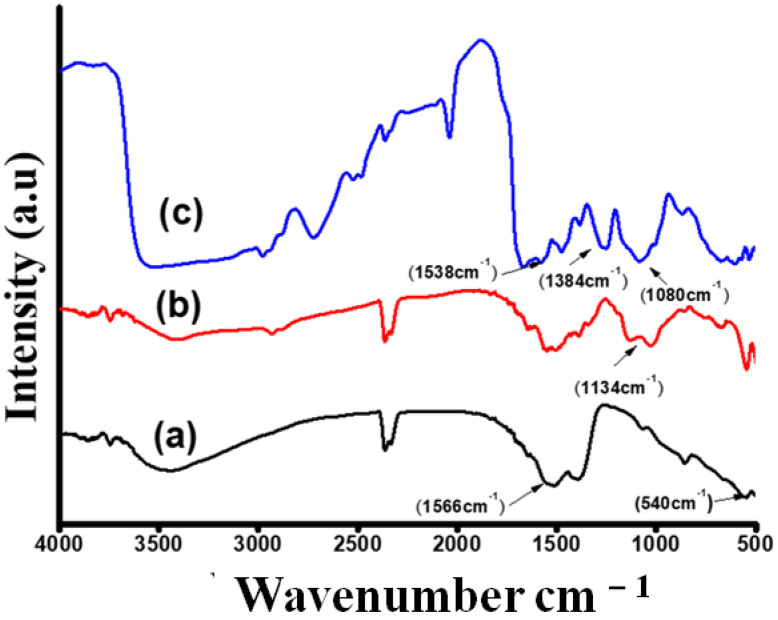
FTIR spectra of (**a**) bare Gd_2_O_3_; (**b**) APTES-Gd_2_O_3_/ITO; (**c**) anti-CT/APTES-Gd_2_O_3_/ITO.

An FTIR study was performed to verify the presence of polar molecules in every critical step (Figure 5). The FTIR spectrum of bare Gd_2_O_3_ NPs showed a typical Gd=O vibration band at around 543 cm^−1^ (Figure 5a), and the absorption bands that appeared at 1496 cm^−1^ and 1394 cm^−1^ corresponded to the C=O band. Those bands were profound and appeared in all spectrums representing Gd_2_O_3_ NPs. This may be due to low-temperature calcination [25,26]. The functionalization of Gd_2_O_3_ NPs by APTES was confirmed by the FTIR spectrum (Figure 5b). In this curve, the broad band between 975 and 1100 cm^−1^ was attributed to the Si–O bond [27]. Peaks within the range of 1490–1580 cm^−1^ corresponded to the deformations of –NH_2_ present in APTES. Furthermore, a broad band presented at 3377 cm^−1^could be described as N−H stretching vibration along with–OH stretching inwater molecules. Immobilization with an antibody was confirmed by FITR (Figure 5c). Apart from the peaks observed for the curve (b), –NH_2_ deformation was confirmed by peaks between 1580–1680 cm^−1^. It could be seen that the intensity of the 1134 cm^−1^ and 1546 cm^−1^ bands (corresponding to –NH_2_ of APTES) decreased considerably in the case of the anti-CT/APTES-Gd_2_O_3_/ITO electrode [23]. Bands appearing near 1384 cm^−1 ^were attributed to the stretching of –COO^−^. Bands appearing within 1400–1420 cm^−1^ were assigned as C–N, which is present in the antibody. The band seen near 1080 cm^−1^ was due to the bending vibration of aliphatic moieties −CH_2_, present in anti-CT. Moreover, IR bands appearing at 1538 cm^−1^ and 1740 cm^−1^ corresponded to amide II and −C=O stretching of the carboxylic group. These results confirmed the covalent attachment of anti-CT to the amino terminal of APTES functionalized Gd_2_O_3_ NPs (Figure 5c) [28].

### 3.2. Electrochemical Characterizations

**Figure 6:**
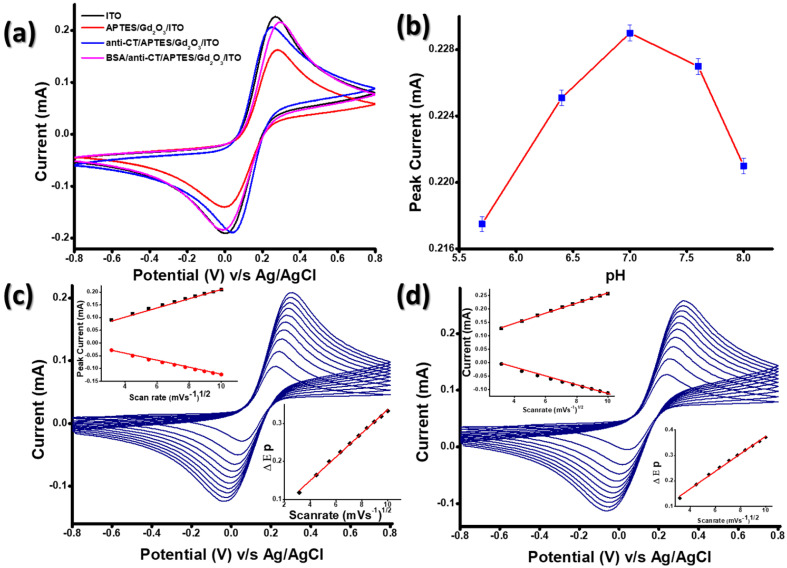
(**a**) CV study of (i) ITO; (ii) APTES-Gd_2_O_3_/ITO; (iii) anti-CT/APTES-Gd_2_O_3_/ITO; (iv) BSA/anti-CT/APTES-Gd_2_O_3_/ITO electrodes in PBS containing[Fe(CN)_6_]^3−/4−^ at a scan rate of 0.05V s^−1^, (**b**) effect of pH on the BSA/anti-CT/APTES-Gd_2_O_3_/ITO immunoelectrode, (**c**) scan rate response of APTES-Gd_2_O_3_/ITO electrode and (**d**) scan rate response of BSA/anti-CT/APTES-Gd_2_O_3_/ITO electrode.

Studies of cyclic voltammetry (CV) conducted on (i) ITO, (ii) APTES-Gd_2_O_3_/ITO, (iii) anti-CT/APTES-Gd_2_O_3_/ITO and (iv)BSA/anti-CT/APTES-Gd_2_O_3_/ITO electrodes are shown in Figure 6a. The magnitude of the anodic current (1.67 mA) for APTES-Gd_2_O_3_/ITO was far less than that of the bare ITO electrode (2.45 mA), which could be due to the deposition of APTES-Gd_2_O_3_ materials onto the ITO surface, which slowed the transfer of electrons generated by redox species [Fe(CN)_6_]^3−/4−^ at the electrode/electrolyte interface as Gd_2_O_3_ NPs are less conductive in nature. However, after the immobilization of anti-CT, the magnitude of the current increased (2.15 mA). This occurred because electron transport between the electrolytes and the electrode surface was promoted by anti-CT molecules that were present on the electrode surface. These findings demonstrated that anti-CT enabled rapid electron transport between electrode/electrolyte interfaces due to the presence of free amine groups.[29,30,31]. Moreover, the amine group of APTES provided a channel by shortening the tunneling distance between the anti-CT and APTES/Gd_2_O_3_/ITO electrode [32]. Because APTES molecules were covalently bound to Gd_2_O_3_ NPs and anti-CT, the APTES/Gd_2_O_3_/ITO electrode surface provided suitable support for the immobilization of biomolecules. Furthermore, there was a slight increase in the magnitude of the current as well as the anodic peak potential after the functionalization of the anti-CT/APTES/Gd_2_O_3_/ITO immunoelectrode with BSA [curve (iv)], which indicates the immobilization of BSA molecules onto the immunoelectrode surface. BSA has been widely used in order to block the activity of non-specific reactive groups [19,20]. This increase in the current of BSA/anti-CT/APTES-Gd_2_O_3_/ITO electrodes was due to a change in the surface charge (energy).

Furthermore, the effect of electrolyte pH on the final sensing electrode, i.e., BSA/anti-CT/APTES-Gd_2_O_3_/ITO, was carried out by performing CV with a scan rate of 0.05 V s^−1^ in PBS containing [Fe(CN)_6_]^3−/4−^ (Figure 6 (b)). The current rose with the increase in the electrolyte pH until pH (7.0), after pH (7.0), the current started decreasing. The maximum peak current (0.229 mA) was obtained at neutral pH, indicating that the BSA/anti-CT/APTES-Gd_2_O_3_/ITO immunoelectrode had high activity at neutral pH. However, with a change in the acidity or basicity of the medium, the microenvironment destroyed the activity of antibodies because of the interaction of H^+^ or OH^−^ ions on the amino acid sequence of antibodies [33].

Moreover, the electrochemical interface kinetics study of APTES-Gd_2_O_3_/ITO (Figure 6c) and BSA/anti-CT/APTES-Gd_2_O_3_/ITO electrodes (Figure 6d) were recorded at different scan rates (10–100mV s^−1^) under similar conditions to determine the changes in the electrochemical properties of the electrodes after antibody immobilization. It was observed that both cathodic (*I_pc_*) and anodic (*I_pa_*) peak currents varied linearly with the scan rate. The linear electrochemical response indicated that the electrochemical reaction was a diffusion-controlled process [23]. The slopes and intercepts were given by the following equations:

(3) $$I_{pc\left(APTES-Gd_{2}O_{3}-ITO\right)=\left[17.23\;\mu A\left(smV^{-1}\right)\times scanrate\left(mVs^{-1}\right)+38.75\;\mu A\right],\;R^{2}=0.998\;}$$

(4) $$I_{pa\left(APTES-Gd_{2}O_{3}-ITO\right)=-\left[13.83\;\mu A\left(smV^{-1}\right)\times scanrate\left(mVs^{-1}\right)-12.49\;\mu A\right],\;R^{2}=0.997\;}$$

(5) $$I_{pc\left(BSA-Anti-CT-APTES-Gd_{2}O_{3}-ITO\right)=\left[18.91\;\mu A\left(smV^{-1}\right)\times scanrate\left(mVs^{-1}\right)+71.42\;\mu A\right],\;R^{2}=0.998\;}$$

(6) $$I_{pa\left(BSA-Anti-CT-APTES-Gd_{2}O_{3}-ITO\right)=-\left[18.91\;\mu A\left(smV^{-1}\right)\times scanrate\left(mVs^{-1}\right)-39.37\;\mu A\right],\;R^{2}=0.995\;}$$

A plot between the difference of cathodic (*E_pc_*) and anodic (*E_pa_*) peak potentials (*∆E_p_* = *E_pc_*− *E_pa_*) and scan rate for APTES-Gd_2_O_3_/ITO and BSA/anti-CT/APTES-Gd_2_O_3_/ITO electrodes exhibited a linear relationship as given by equations (vii) and (viii). A good linear fitting suggested a facile electron transfer from the medium to the electrode.

(7) $$\Delta E_{p\left(APTES-Gd_{2}O_{3}-ITO\right)=\left[0.031\;V\left(smV^{-1}\right)\times scanrate\left(mVs^{-1}\right)+2.62\;mV\right],\;R^{2}=0.99\;}$$

(8) $$\Delta E_{p\left(BSA-Anti-CT-APTES-Gd_{2}O_{3}-ITO\right)=\left[0.034\;V\left(smV^{-1}\right)\times scanrate\left(mVs^{-1}\right)+32.05\;mV\right],\;R^{2}=0.99\;}$$

The value of diffusion coefficient (*D*) was estimated using the Randles–Sevcik equation:

(9) $$I_{p}=\left(2.69\times 10^{5}\right)n^{\frac{3}{2}}AD^{\frac{1}{2}}C\nu^{\frac{1}{2}}$$

where $I_{p}$ is the peak current of the electrode, *n* is the number of electrons transferred (*n* = 1), A is the working electrode surface area, D is the diffusion coefficient, C is the concentration of the redox species [Fe (CN)_6_]^3−/4−^ and υ is the scan rate (0.05 V s^−1^). The diffusion constant was estimated to be 8.876 × 10^−12^ cm^2^ s^−1^ BSA/anti-CT/APTES-Gd_2_O_3_/ITO immunoelectrode.

Since electrochemical sensing is also affected by surface charge, the concentration of ionic species at the electrode surface plays an important role. Thus, the surface concentration of ionic species of these electrodes was calculated using the Brown–Anson model [19]:

(10) $$E_{p}=\frac{n^{2}F^{2}I^{*}A\nu }{4RT}$$

where *n* is the number of electrons (here, it is 1), *F* is the Faraday constant (96485.34 C mol^−1^), I^*^ is the surface concentration of ionic species at the immuno-electrode (mol cm^−2^), *T* is 298 K, and *I_p_/V* is the slope of the calibration plot (scan rate value), *A* is the surface area of the electrode (0.25 cm^2^) and *R* is the gas constant.

The surface concentration of APTES-Gd_2_O_3_/ITO and BSA/anti-CT/APTES-Gd_2_O_3_/ITO immunoelectrode was found to be 1.87 × 10^−8^ and 1.91 × 10^−8^ M cm^−2^, respectively, indicating that the APTES-Gd_2_O_3_/ITO electrode offered an increased electroactive surface area for stacking antibodies (anti-CT). Nevertheless, after immobilization of the anti-CT, the surface concentration changed, indicating the presence of anti-CT and BSA on the BSA/anti-CT/APTES-Gd_2_O_3_/ITO electrode surface with multilayer coverage.

The value of the diverse electron transfer rate constant (*K_s_*) found for the APTES-Gd_2_O_3_/ITO and BSA/anti-CT/APTES-Gd_2_O_3_/ITO electrode was calculated to be 0.12 and 0.15 s^−1^ according to the model of the Laviron equation[19].

(11) $$K_{s}=\frac{mnF\nu }{RT}$$

where *m* is the inter-peak separation (0.12 and 0.14 V, respectively). A satisfactory value of *K_s_* was obtained in the case of the APTES-Gd_2_O_3_/ITO electrode as compared to the BSA/anti-CT/APTES-Gd_2_O_3_/ITO immunoelectrode due to the absence of anti-CT and BSA. These above electrochemical characterizations showed that the sensor composed of the BSA/anti-CT/APTES-Gd_2_O_3_/ITO immunoelectrode assisted in the charge transfer; therefore, it is suitable for electrochemical sensing applications.

### 3.3. Electrochemical Response Studies

**Figure 7:**
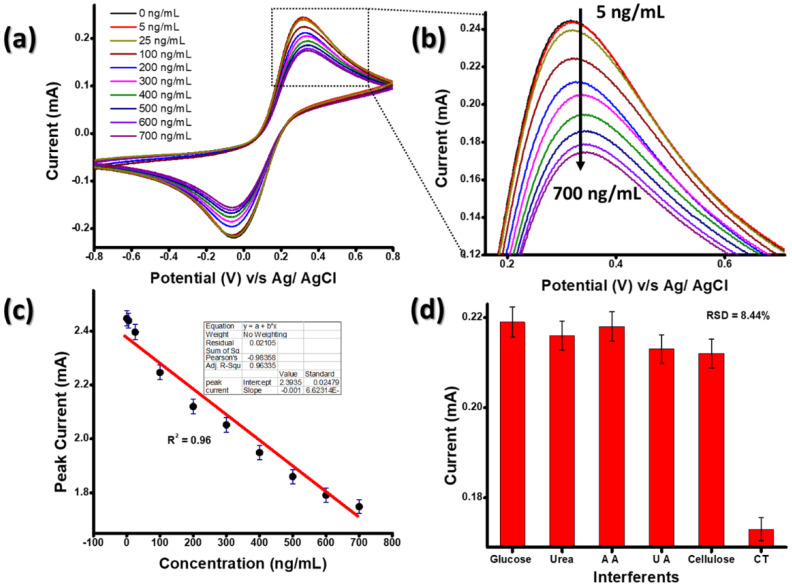
(**a**,**b**) The electrochemical response study of BSA/anti-CT/APTES-Gd_2_O_3_/ITO immunoelectrode for different concentration of CT, (**c**) the calibration plot and (**d**) the interference study of BSA/anti-CT/APTES-Gd_2_O_3_/ITO.

The electrochemical response of the BSA/anti-CT/APTES-Gd_2_O_3_/ITO immunoelectrode was measured as a function of CT concentration (5–700 ng mL^−1^) in PBS containing [Fe (CN)_6_]^−3/−4^ at a scan rate of 0.05 V s^−1^ using the CV technique (Figure 7). All the measurements were repeated three times for each concentration. It was seen that the magnitude of the current decreased with the increase in the concentration of CT varying from 5 to 700 ng mL^−1^.

This electrochemical response could be ascribed to the formation of an immune complex (CT bind with the BSA/anti-CT/APTES-Gd_2_O_3_/ITO immunoelectrode), which hinders the electron transfer between the immunoelectrode and electrolytes (Figure 7b) [23].

The linear curve was plotted between the anodic peak current and CT concentration (Figure 7c). The linear regression coefficient (R^2^) for the linear curve obtained was 0.96. The sensitivity of the immunoelectrode was calculated to be 8.37 mA ng^−1^ mL cm^−1^. The value of the LOD was 1.48 ng mL^−1^, as calculated from the equation of 3σ/m, where σ is the standard error of the linear plot and m is the slope. The BSA/anti-CT/APTES/Gd_2_O_3_/ITO immunoelectrode exhibited excellent sensitivity in other works reported in the literature for *Vibrio cholerae* detection. Additionally, this immunoelectrode has almost the lowest LOD. Thus, the APTES-Gd_2_O_3_ NP-based immunosensor is an excellent sensor for cholera detection. It is worth mentioning that the immunosensor developed using Gd_2_O_3_ NPs is comparable with the previously reported method of CT detection. Table 1 shows the biosensing properties of previously reported biosensing platforms for CT detection.

The selectivity of the immunoelectrode is determined by changes in peak currents in the presence of different interfering analytes (Figure 7d). This study was performed on the BSA/anti-CT/APTES-Gd_2_O_3_/ITO immunoelectrode using various potential interfering substances in the physiological range of glucose (≤13.6 mM L^−1^), urea (≤14 mM L^−1^), uric acid (UA) (5 mg dL^−1^), ascorbic acid (2 mM) (AA), cellulose (1.1 mM) and CT (700 ng mL^−1^). The fabricated immunoelectrode showed the highest specificity for CT in the presence of different interfering analytes with a relative standard deviation (RSD) of 8.44%, indicating the clinical importance of the designed sensor in cholera detection.

## 4. Conclusions

Gd_2_O_3_ NPs were synthesized via the microwave method and functionalized with APTES to generate an -NH_2_ group on the surface. Gd_2_O_3_ NPs were thoroughly characterized by TEM, XRD, FTIR and UV/Vis techniques to ascertain their physical properties. An APTES/Gd_2_O_3_/ITO film was prepared with an electrophoretic deposition technique onto ITO. Anti-CT and BSA were immobilized to fabricate the BSA/anti-CT/APTES/Gd_2_O_3_/ITO immunoelectrode for the detection of CT. The immunosensor exhibits a wide detection range of 5 to 700 ng mL^−1^ and LOD of 1.48 ng mL^−1^with a sensitivity 8.37 mA ng^−1^ mL cm^−2^, which shows the clinical significance of the designed sensor probe. The immunoelectrode showed excellent selectivity towards *Vibrio cholerae*. Moreover, the proposed biosensor can be utilized to manufacture non-invasive biosensors for cholera detection.

## Figures and Tables

**Table 1:** Comparison of biosensing properties of the BSA/anti-CT/APTES/Gd_2_O_3_/ITO immunoelectrode reported in the literature.

Electrode/Immobilization	Transducer	Biomolecules	Detection Range	Low Detection Range	Sensitivity	References
Au Electrode	DNA biosensors	ss-DNA	100–500 ng mL^−1^	100 ng mL^−1^	0.027 μA/ng cm^−2^	[12]
RGO-TiO_2_/ITO	Electrochemical impedance spectroscopy	Antibodies	10–450 ng mL^−1^	0.15 ng mL^−1^	21.8 × 10^−3^μF/ng L^−1^cm^−2^	[13]
BSA/Ab/CA-Fe_3_O_4_/ITO	Electrochemical Impedance spectroscopy	Antibodies	12.5–500 ng mL^−1^	0.32 ng mL^−1^	0.03 Ω/ng mL^−1^ cm^−2^	[14]
BSA/Ab/GelA-ZrO_2_/ITO	Electrochemical impedance spectroscopy	Antibodies	50–400 ng mL^−1^	0.74 ng mL^−1^	0.03 Ω/ng mL^−1^cm^−2^	[15]
BSA/Ab/OA-Fe_3_O_4_/ITO	Electrochemical impedance spectroscopy	Antibodies	12.5–500 ng mL^−1^	0.5 ng mL^−1^	0.1 Ω/ng mL^−1^cm^−2^	[28]
NiO/ITO nanowires	Electrochemical impedance spectroscopy	Antibodies	37–350 ng mL^−1^	0.553 ng mL^−1^	11.12 Ω/ng mL^−1^ cm^−2^	[29]
Nanocoaxial array	Differential Pulse Voltammetry	ELISA	10 ng mL^−1^–1 μg mL^−1^	2 ng mL^−1^	-------	[30]
IDUA electrodes	Microfluidic assay	Antibodies	0–100 ng mL^−1^	31.7ng mL^−1^	--------	[31]
Graphene Nano sheets with incorporated lipid films	Potentiometric	GM1	10 × 10^−9^M to 10 × 10^−6^M	1 nM	∼60 mV/decade	[32]
BSA/anti-CT/APTES/Gd_2_O_3_/ ITO	Cyclic Voltammetry	Antibodies	5–700 ng mL^−1^	1.48 ng mL^−1^	8.37 mA ng^−1^mL cm^−2^	Present Work

## Data Availability

Not applicable.
